# Development of the Adult Pandemic Attitude Scale (A-PAS)

**DOI:** 10.3390/ijerph18126311

**Published:** 2021-06-10

**Authors:** Mihyeon Seong, Juyoung Park, Soojin Chung, Sohyune Sok

**Affiliations:** 1 Department of Nursing, Changshin University, Changwon-si 51352, Korea; mihyeon0624@cs.ac.kr; 2 Department of Nursing, Kunjang University, Gunsan 54045, Korea; jypark@kunjang.ac.kr; 3 Department of Nursing, Graduate School, Kyung Hee University, Seoul 02447, Korea; nijoosgnuhc@khu.ac.kr; 4 College of Nursing Science, Kyung Hee University, Seoul 02447, Korea

**Keywords:** instrument, development, attitude, pandemic, A-PAS

## Abstract

This study aimed to develop an instrument for measuring the attitudes that reflect the characteristics of the pandemic (Adult Pandemic Attitude Scale (A-PAS)) and verifying its validity and reliability. This study used a methodological research design and was conducted with a development step and an evaluation step. The development step included development of preliminary items, content validity, face validity, and preliminary investigation. The evaluation step included item analysis, construct validity, convergent validity, discriminant validity, criterion validity, factor naming, reliability, and completion of the final instrument. The A-PAS developed in this study consisted of a total of 20 items in five dimensions. The internal consistency of 20 items of the A-PAS, Cronbach’s α was 0.92 for 20 items, Cronbach’s α for each factor, a subscale of instrument, was 0.61~0.87 and Raykov’s *p* coefficient of each factor, which is a subscale of the tool, was found to be 0.60 to 0.88. Analysis of construct validity showed the results as follows: χ^2^ (*p*) = 134.05 (*p* < 0.001), RMSEA = 0.02, RMR = 0.02, GFI = 0.94, CFI = 0.99. The study findings suggest that the developed instrument can be utilized to measure the attitudes of adults toward pandemics, and reflect the reality of the pandemic situation. The outcomes can be used as valuable data for intervention, prevention activities, and policy preparation. The instrument will be applied in the event of a pandemic, such as COVID-19, and will be helpful in promoting the health of the people.

## 1. Introduction

The World Health Organization (WHO) reported the first outbreak of the coronavirus disease-2019 (COVID-19), caused by severe acute respiratory syndrome coronavirus 2 (SARS-CoV-2), in December 2019 [1,2]. SARS-CoV-2, which broke out in 2019, has become a major public health problem worldwide, causing great fear in people and is still ongoing as a pandemic in 2021 [2,3]. However, since the outbreak of H1N1 influenza in 2009, health-related ministries in each country have been paying special attention to preparing strategies to prepare for a pandemic and prevent its spread [4]. Although lessons from the past have provided various solutions, they are insufficient to overcome the present reality. Even if well prepared with vaccinations, a pandemic such as the influenza, that occurs every year, can cause considerable damage due to unpredictable factors. Moreover, experts recognized that another pandemic was inevitable [5].

Along with the potential risk of a pandemic, uncertainty can lead to confusion in communities and countries [6], this unprecedented time causes suffering and weakens the minds of many people, leading to health problems and mental health problems [6,7,8]. In response to this, some countries enforced quarantine by closing the borders and airports, disrupting trade, stock markets, and productivity, and directly affecting global security [9]. As such, the current global pandemic of COVID-19 has adversely affected the global economy due to the closure of several regions [10]. Nationally, the financial penalties of the people, due to the increase in telecommuting, delay of examinations due to the closure of educational institutions, and uncertainty, caused many problems [11]. These problems may cause stress, and new diseases such as COVID-19 can cause confusion, anxiety, and fear [1]. Fear, in particular, can lead to social stigma, thereby resulting in a situation where people hide the disease and do not receive medical care [1]. Due to the pandemic, people are under great stress, so somatization symptoms appear, and even medical staff can solve mental health problems [12,13,14,15]. It is also necessary to address complications such as mental health, central nervous system symptoms, and respiratory problems for people with COVID-19, and to consider potential disability risks [16,17]. As such, results of mental health, health, and well-being challenged by COVID-19 are likely to be long lasting [18]. Therefore, we need to support people to better prepare, and we will need to deal with another new infectious disease in the future.

Since the outbreak of the COVID-19 pandemic, governments, media, medical staff, and other social officials have appealed to the public to refrain from public gatherings (e.g., religious ceremonies, family events, meetings, and school classes) in order to prevent the spread of COVID-19 around the world. Despite these efforts, many people disregarded national responses, such as travel restrictions and social distancing [19]. The government’s efforts to control and manage infections are affected by the attitudes, knowledge, and perceptions of communities and individuals [20,21]. In particular, problems with people’s attitudes lead to pain and panic [10]. The effectiveness of public health policies will depend on how well the general public agrees to and adheres to the rules, and public attitudes are more likely to adopt the rapid behavior changes needed to address the challenges posed by COVID-19 [22]. These individual actions are critical to contain the spread of COVID-19 [23]. Although there are many studies on the epidemiology, causes, and clinical characteristics of COVID-19, research on the attitudes and knowledge of the general public about the novel disease is relatively insufficient [24].

In order to cope with a pandemic situation, not only medical personnel but everyone should work together [21], so a rationale for immediate intervention to strengthen psychological resilience and strengthen the capacity of the medical system should be prepared [15,25]. Therefore, it is necessary to understand the attitudes of the general public in a pandemic situation, and to promote appropriate behavioral patterns for the situation.

Attitude, as a major factor determining behavior along with knowledge [26], refers to an attempt to specifically express individual emotions and tendencies of thinking [27]. It includes cognitive, affective, and behavioral factors [28], and it is known that attitudes toward health appear as tentative behaviors and affect health behavior practices [29]. The Knowledge-Attitude-Practice (KAP) model is mainly used to understand people’s attitudes toward the pandemic, and a high level of knowledge, positive attitude, and accurate practice are essential to controlling the spread of COVID-19 [21]. However, this tool has a limitation in that the sensitivity is not high as the characteristics of the pandemic are not melted.

Therefore, the aims of this study were to develop an instrument for measuring the attitudes that reflect the characteristics of the pandemic (Adult Pandemic Attitude Scale (A-PAS)) and verifying its validity and reliability.

## 2. Material and Methods

### 2.1. Study Design

This study was a methodological research that developed and evaluated the instrument to measure the attitudes that reflect the characteristics of the pandemic (Adult Pandemic Attitude Scale (A-PAS)). This study was conducted with a development step and an evaluation step (Table 1).

### 2.2. Instrument Development Process

#### 2.2.1. Composition of Preliminary Items

The instrument development step of this study consisted of the preparation of preliminary items, verification of content validity and face validity, and preliminary investigation. For the literature on pandemics, diseases, and health attitudes of the preliminary items, the databases of MEDLINE, CINAHL, PubMed, and Google Scholar were used to review domestic and international literature suitable for the research topic. In addition, the contents of individual interview items were organized according to the situation of the experts and the general public, and in-depth interviews were conducted with 3 experts and 7 ordinary adults using semi-structured and open methods. Subjects for in-depth interviews were (1) those aged 20 years or older, (2) those without serious physical or mental problems, (3) those who can communicate and understand the item contents, and (4) those who understand the purpose of this study and agree to participate in the study. A total of 57 items were derived from an integrated analysis of the literature review and in-depth interview. Following a review of the initial preliminary items, 31 preliminary items were selected through revision and supplementation.

#### 2.2.2. Determination of the Scale of the Instrument

For the scale of this instrument, the Likert scale was used. It is most often used in the attitude measurement for quantification, and it can determine the number of response categories according to the phenomena to be investigated and the research purpose [30]. As for the response category of the Likert scale, the most reliable are the 5-point scale or 6-point scale. The use of the 5-point Likert scale was determined based on the evidence that a distribution error may occur when a respondent with a neutral opinion on a question forcibly selects a negative or positive opinion [30,31].

#### 2.2.3. Content Validity

In this study, the content validity of 31 preliminary items was verified. Based on the evidence that it is preferable to have a group that consists of 3–10 experts for the verification of content validity [32], the opinions of 10 experts interested in pandemic attitudes were collected. The 10 experts included 5 nursing professors, 1 medical professor, 3 persons with Ph.D. in public health or nursing, and 1 psychology professor. The content validity was tested by calculating the Scale-Content Validity Index/Average (S-CVI/Ave) with the average of the Item-Content Validity Index (I-CVI) [33,34]. In the verification of content validity, the participants were guided on rating the validity of the item in order to measure the pandemic attitude with a 4-point Likert scale (1 point = not valid at all; 2 points = not valid; 3 points = valid; and 4 points = very valid). For the interpretation of the test results of content validity by experts, it was judged to be valid as an item to measure the pandemic attitude when the CVI value for each item is 0.80 or higher. If the content of the item was considered to be ambiguous or inappropriate when the experts responded, they were allowed to enter opinions for revisions in order to revise or supplement the expression of the item.

As a result, I-CVI ranged from 0.63 to 1.00, and the items with a content validity index of 0.80 or higher were selected; S-CVI/Ave was 0.84, thus meeting the content validity criteria [32].

#### 2.2.4. Face Validity

The verification of face validity was conducted in two rounds from 23 January 2021 to 25 January 2021, based on 27 items that were finalized through the verification of content validity. In the first round, the items were verified by a total of 3 experts (1 professor of infectious medicine, 1 professor of respiratory medicine, and 1 field leader in community nursing). In the second round, the items were verified by a total of 2 persons (1 adult male and 1 adult female). This process resulted in the removal of two items, the correction of some of the items with awkward expressions and expressions that did not fit the grammar, and the finalization of 25 items.

#### 2.2.5. Preliminary Investigation

For the finalized 25 items, a preliminary investigation was conducted on 10 adults who were selected through convenience sampling. The expression, arrangement, format, appropriateness of the items, and required time for the survey were examined.

### 2.3. Instrument Evaluation Step

#### 2.3.1. Study Subjects

It was not necessary to apply strict criteria to the sample size by comprehensively considering the size at least 5 times the total number of items [33], the number of measured variables, the number of factors, and factor loadings in the factor analysis. However, the sample size was determined in consideration of the criteria that at least 4 times the number of items, or 200 or more, were desirable [35], and the opinion that 150–200 people for the exploratory factor analysis, and 130–200 people for the confirmatory factor analysis were required [36]. In this study, 150 subjects were selected for the exploratory factor analysis and 200 subjects were selected for the confirmatory factor analysis; therefore, 170 subjects for the exploratory factor analysis and 220 subjects for the confirmatory factor analysis were selected as the target number of subjects, considering an approximate 10% dropout rate. The inclusion criterion of study participants was the general public aged 20 years or older.

#### 2.3.2. Data Collection and Ethical Considerations

Prior to performing the research, it was reviewed and approved by the Institutional Research Board of C University located in C city. The data were collected from 28 January 2021 to 8 February 2021. As the COVID-19 pandemic continued, it became difficult to conduct a face-to-face survey, so a non-face-to-face survey was conducted using an Internet platform. Recruitment announcements were posted through the Internet portal site, and the information sheet, which included the purpose, procedure, and method of the study, data confidentiality, the research data management method, the possibility of withdrawal, and the contact information of the researcher, was provided in the posts. The survey was conducted only when the participant read and reviewed the information sheet sufficiently, and voluntarily agreed to participate in the study. The required time to complete the questionnaire was about 5 min. All 390 questionnaires were collected, and all of them were used for the final analysis.

#### 2.3.3. Data Analysis Method

The data collected for the item analysis, validity, and reliability test of the developed instrument were analyzed using the IBM SPSS Statistics version 20.0 (IBM, Armonk, NY, USA) and IBM SPSS AMOS Statistics version 23.0 statistical programs (IBM, Armonk, NY, USA).

For the general characteristics of the study participants, frequency, percentage, mean, and standard deviation were calculated by using descriptive statistics [36]. In order to verify the construct validity of the measurement instrument, exploratory factor analysis and confirmatory factor analysis were used. Kaiser-Meyer-Olkin (KMO) values were calculated, and Bartlett’s test of sphericity was performed for the exploratory factor analysis to verify the construct validity. As an exploratory factor analysis method using the principal component analysis and varimax rotation, factors were extracted based on an eigenvalue of 1.00 or higher and, for the factor loading, a reference of >0.40 was applied [37]. Confirmatory factor analysis can evaluate the construct validity of new constructs for structural equation models based on factors classified through exploratory factor analysis, quantitatively assess their quality, and provide additional evidence of measurement validity [36]. χ^2^, CMIN/df, approximate root mean square error (RMSEA), root mean square residual (RMR), standardized root mean square residual (SRMR), goodness-of-fit index (GFI), incremental fit index (IFI), Tucker-Lewis Index (TLI), and Compare Fit Index (CFI) [36], the significance was also verified by applying >0.40 for the factor loading of each item [37]. The convergent validity of A-PAS was verified using standardized factor loading, critical ratio (C.R.), construct reliability (CR), and average variance extracted (AVE). For the discriminant validity, it was considered to have discriminant validity if the correlation coefficient between factors did not exceed 0.90 and the AVE of the latent variable was greater than the square of the correlation coefficient between the latent variables [38,39]. For the criterion validity, concurrent validity was verified by using the Adult Pandemic Attitude Scale (A-PAS) developed in this study and the Health Attitude Scale of Torabi et al. [40], which has been widely used in the past. The Pearson correlation coefficient for the measured values of the two instruments were measured. The reason the Health Attitude Scale was selected as the criterion for testing the criterion validity is that there are a few instruments to measure the psychosocial aspects of people about pandemics. In addition, the pandemic attitude can act as a new predictor of people’s health behavior in a pandemic situation. As a result, it was selected by referring to the method of selecting the criterion validity of previous study [41]. The author’s consent was obtained for the use of the Health Attitude Scale, and the instrument of Kim [42], which was modified and supplemented after being translated into Korean, was used. The instrument is a 5-point Likert scale consisting of 5 items about feelings on health, 5 items about belief in disease prevention and a healthy lifestyle, and 5 items about health behavior with the intention to become healthier. The instrument consisted of 1 point ‘Strongly disagree’ to 5 points ‘Strongly agree’. At the time of development, Cronbach’s α of the instrument was 0.88.

Reliability was confirmed by internal consistency reliability (Cronbach’s α coefficient and Raykov’s *p* coefficient) in order to confirm the homogeneity among the items of the instrument developed in this study.

## 3. Results

### 3.1. Development Step

#### 3.1.1. Development of Preliminary Items

For the 57 preliminary items derived from the literature review and in-depth interview data, sentences suitable for measuring the pandemic attitude of adults were selected. The first preliminary items were independently selected, and 41 preliminary items were selected, excluding those with disagreements. For the second preliminary item selection, the final 31 preliminary items were finalized through an in-depth review of the content for each item, excluding duplicate content and revising similar and ambiguous sentences.

#### 3.1.2. Content Validity

As a result of the content validity verification by experts, I-CVI ranged from 0.63 to 1.00, and four items with a CVI value of less than 0.80 were eliminated. In addition, the questionnaire was condensed into 27 items. S-CVI/Ave met the content validity criterion with 0.84 [32]. In regards to the converged items, opinions on the revision of the items were reflected, such as changing an item from ‘I have to wash my hands more often when there is a pandemic’ to ‘I think I should pay more attention to personal hygiene when there is a pandemic’.

#### 3.1.3. Face Validity

Based on the 27 items finalized through the verification of content validity, face validity was verified in two rounds. In the first round, three experts (one professor of infectious medicine, one professor of respiratory medicine, and one field leader in community nursing) participated in the verification. As a result, the items ‘I know that when I cough or sneeze, I should cover it with my sleeves’ and ‘I think if I touch objects that people touch a lot (e.g., bus handles, door handles, handrails, etc.), I can get infected’ were removed. In the second round, two persons (one adult male and one adult female) participated in the verification, and 25 items were finalized by revising awkward expressions and expressions that did not fit the grammar in some of the items.

#### 3.1.4. Preliminary Investigation

As a result of the analysis of the preliminary instrument through the preliminary investigation, the subjects of the preliminary investigation were ordinary adults living in G province, Korea, and the average age was 39.60 ± 13.66 years old. It took approximately 5 min to fill out the questionnaire, and the internal reliability of the preliminary instrument Cronbach’s α was 0.90.

### 3.2. Evaluation Step

#### 3.2.1. General Characteristics of the Study Participants

Regarding general characteristics of the study participants, item analysis and exploratory factor analysis were performed on 170 subjects in order to evaluate the reliability and validity of the initial pandemic attitude scale, and 220 subjects were used for the confirmatory factor analysis. There was no statistically significant difference in the general characteristics between the two data. General characteristics of the study participants are shown in Table 2.

#### 3.2.2. Item Analysis

The correlation coefficient between the item and the total score were calculated, and the reference of 0.30 was applied [43]. There was no item with a correlation coefficient of less than 0.30 between each item and the total score for 25 preliminary items (Table 3).

#### 3.2.3. Construct Validity

As a result of confirming the fit for factor analysis of 25 items, the KMO value was 0.90, and the χ^2^ value was 2016.86 *(p* < 0.001) as a result of Bartlett’s test of sphericity, thus confirming the sampling adequacy. Through the exploratory factor analysis, five factors with an eigenvalue of 1.0 or higher were extracted, which accounted for 60.6% of the total variance. As a result of the exploratory factor analysis, the factor loadings of all 25 items were >0.40.

The model fit was verified by performing a confirmatory factor analysis on the five factors classified through exploratory factor analysis, and (Model 1) was constructed. As a result of examining the standardized factor loadings for 25 items, five items with a standardized factor loading of lower than 0.50, lowering the overall model fit, were removed to construct (Model 2): item no. 1, ‘I think I could infect others’; item no. 8, ‘I think a pandemic can occur at any time, and we should prepare for it’; item no. 11, ‘I think the country or government has limitations in preventing infection once the pandemic begins, so I should be careful first’; item no. 18, ‘I will actively participate if there is an education about pandemics’; and item no. 25, ‘I’m very careful because I’m afraid I’ll be stigmatized because of the pandemic’. The standardized factor loadings of 20 items in (Model 2), excluding 5 items, were found to be 0.62~0.89 (Table 2).

As a result of the normality test for the confirmatory factor analysis in (Model 2), the skewness was −1.74 to −0.14, and the kurtosis was −0.74 to 4.52, thereby satisfying the univariate normality assumption, as it did not exceed 3 of the absolute value of skewness and 10 of the kurtosis [44]. In the multivariate normality test, the multivariate kurtosis index was 166.08 and the critical value was 41.52, thereby exceeding the threshold criterion of 5.99, which did not satisfy the multivariate normality assumption [44]. However, for the analysis of the data that violated the assumption of multivariate normality in this study, the significance of the model path was verified through the regression coefficient and *P* value by using the bootstrapping method for parameter estimation and effect analysis [45]. The χ^2^ value was 395.90 (df = 160, *p* < 0.001), and the normed chi-square value was 2.47 in this study, thereby satisfying the criteria that if it was less than 3, it was acceptable [39].

Other model fit indices were absolute fit indices RMSEA = 0.08, GFI = 0.84, RMR = 0.03, and SRMR = 0.06, and incremental fit indices CFI = 0.89, IFI = 0.89, Normed Fit Index NFI = 0.83, and TLI= 0.89, thereby not meeting the criteria. Therefore, after setting the correlation between the measurement errors of exogenous latent variables, (Model 3) was constructed, and the results of analyzing the fitness of (Model 3) are as follows. The χ^2^ value was 134.05 (df = 120, *p* < 0.001), and the normed chi-square value was lowered to 1.12, thereby satisfying the criteria that if it was less than 3, it was acceptable, and if it was less than 2, it was good [26]. The Goodness of Fit indices RMSEA = 0.02, GFI = 0.94, RMR = 0.02, and SRMR = 0.04, and incremental fit indices CFI = 0.99, IFI = 0.99, NFI = 0.94, and TLI = 0.99, thereby showing Goodness of Fit indices of greater than or equal to good [39]. Therefore, the construct validity of the A-PAS consisting of five dimensions of 20 items was confirmed (Table 4).

#### 3.2.4. Convergent Validity and Discriminant Validity

Convergent validity and discriminant validity were used to verify the validity of the concept of the tool consisting of 20 items of five factors. As a result of checking the mean variance extraction (AVE) for the five factors of the tool for intensive validation, the concentrated validity of A-PAS in the mean variance sample study was determined by the standardized factor load, mean variance extraction index, and concept. This was verified based on the reliability value (Table 5). The standardized factor loading of 20 items does not have an exact criterion to determine the significance of the factor loading of each question, but it can be said that it is significant if it is 0.40 or more [33]. The variance extraction index (AVE) and construct reliability (CR) are judged to be appropriate if they are above the threshold values of 0.50 and 0.70, respectively [46]. In this study, the mean variance extraction index (AVE) was 0.54 to 0.73, the critical ratio (C.R.) was 5.75 to 11.11, and the construct reliability was 0.70 to 0.92, confirming that the tool satisfies all the criteria for centralized validity [39].

In addition, in this study, in order to verify the discriminant validity of the A-PAS, each AVE value between the two constructs was compared with the squared value of the correlation coefficient between the two constructs (Φ2), and the AVE value was the square of the correlation coefficient. If it is greater than the value, it is considered to satisfy the criterion of discriminant validity [38,39]. In this study, the AVE value of each factor was found to be greater than the squared value of the correlation coefficient. As found in the process of checking whether the calculated value of the correlation coefficient (Φ) ± (2X standard error) between the constituent concepts did not include 1, all four factors did not include 1, therefore discriminant validity was secured. As for rule validity, all relationships between factors are in the positive (+) direction, and all of them were found to be statistically significant, so law validity was also secured (Table 6).

#### 3.2.5. Criterion Validity

In this study, in order to confirm the criterion validity of the A-PAS, the relationship with the Health Attitude Scale was analyzed with Pearson correlation coefficient. As a result, there was a statistically significant positive correlation with the health attitude (r = 0.28, *p* < 0.001), thereby securing the criterion validity [47].

#### 3.2.6. Factor Naming

The factors were named, centering the items with a higher factor loading. Factor 1 was named ‘personal belief attitude’ with eight items. Factor 2 was named ‘knowledge attitude’ with five items. Factor 3 was named ‘normative attitude’ with two items. Factor 4 was named ‘emotional attitude’ with three items. Factor 5 was named ‘managerial attitude’ with two items (Table 4).

#### 3.2.7. Reliability

To measure reliability in this study, Cronbach’s α and Raykov’s *p* coefficient were utilized. As a result of checking the internal consistency of 20 items of the A-PAS, Cronbach’s α = 0.92, and Cronbach’s α for each factor, a subscale of the instrument was 0.61~0.87, which is in line with the criteria that can be recognized as a newly developed tool [47]. However, Raykov’s *p* coefficient was obtained to replace the problem of reliability for a factor consisting of two items. Raykov’s *p* coefficient of each factor, which is a subscale of the tool, was found to be 0.60 to 0.88. Raykov’s *p* coefficient is presented as a reliability that can cope with Cronbach’s α, and it is accepted that 0.60 or more is acceptable [45,48].

#### 3.2.8. Completion of the Final Instrument

The Adult Pandemic Attitude Scale (A-PAS), which could measure the attitude of ordinary adults toward the pandemic, was finally constructed after verifying the validity and reliability of the instrument. It consisted of a total of 20 items: eight items for ‘personal belief attitude’; five items for ‘knowledge attitude’; two items for ‘normative attitude’; three items for ‘emotional attitude’; and two items for ‘managerial attitude’. The instrument uses a 5-point Likert scale, and the total score range of the instrument is 20 to 100 points. The higher the score, the higher the level of attitude toward the pandemic (Table 7).

## 4. Discussion

The instrument for measuring the attitudes of adults toward pandemics developed in this study consisted of a total of 20 items in five dimensions.

In order to secure content validity during the instrument development process, content validity and face validity were verified on experts and adults, opinions for item revision were collected, and a preliminary investigation was conducted on the subjects. In order to verify the construct validity of the instrument, exploratory factor analysis was performed in the first instrument evaluation process, and analysis was performed with another sample to secure the cross-validity of the confirmatory factor analysis in the second instrument evaluation process. The internal consistency reliability of this instrument was high; however, the confirmatory factor analysis showed that the standardized factor loading was low, and the model fit was slightly less than the acceptance criterion. The final model was confirmed by establishing a correlation between the measurement errors of the exogenous latent variables. In addition, validity was secured in the verification of convergent validity, discriminant validity, and criterion validity.

As a result of the exploratory factor analysis, the first evaluation process of the instrument, it was classified into 5fivefactors. The first factor included cognitive elements such as ‘I think I should pay more attention to personal hygiene when there is a pandemic’; ‘The idea that it is ok for me alone to break the rules can be problematic’; and ‘I think the pandemic can have a serious impact on society overall’. This supports the opinion of Petty et al. [49], who suggested that cognition makes a difference in attitude formation and can be a variable influencing attitude toward behavior. For example, based on the health belief model and the theory of planned behavior, attitudes played an important mediating role in people’s perception of the risk of pandemic influencing their intention to conduct untact tourism [50]. Attitude includes all cognitive, emotional, and behavioral elements. A cognitive element means knowledge or belief about an object, an emotional element indicating likes or dislikes about an object, and a behavioral element that indicates behavioral intentions or actions toward the object. As it is composed of elements, and is formed and manifested as an experience of an influence that directly or indirectly affects a person’s response, [50,51], the results of this study show that cognitive improvement and education must be included when developing interventions that can change behavior based on attitudes toward pandemics.

The second factor of this instrument was composed of items such as ‘When a pandemic occurs, I try to obtain information through the mass media’ and ‘I advise my family about infection and urge them to be careful when a pandemic occurs’. This supports the opinion of Lee et al. [52], who said that sufficient knowledge must be provided through information related to pandemics in order to induce a desirable behavior, and that such knowledge is an important variable that determines behavior. Regarding pandemics, the general public has a reasonable level of knowledge, but is still uncertain about obtaining specific information regarding the spread or prevention of the disease. Thus, it is important to increase knowledge through information provision in order to raise awareness of personal hygiene or safety methods, and reduce fear of the disease [53]. This is due to knowledge, learning content, and experiences affect attitudes, and a lack thereof potentially resulting in negative attitudes [53,54]. As attitudes are formed through knowledge [55], programs that can cultivate positive attitudes for pandemic prevention or health promotion should be prepared based on the knowledge dimension identified in this study.

The third factor of this instrument is the normative dimension including ‘I get angry when I see people who ignore government guidelines’ and ‘I follow the guidelines provided by the government precisely because fake news or misinformation adversely affects pandemic control’. Norms refer to universal principles of behavior that we must follow [56]. They affect perception in the relationship of existence and consciousness, and influence morality and discrimination that occur in human social life [57]. Norms also refer to the fact that an individual expects others to behave in a moral and appropriate way, and others expect that person to do the same [58]. In this study, the reason why the normative dimension appeared as a constituent element is that a pandemic is not an individual problem. Therefore, it is thought that the attitude toward meeting the expectations of others and vice versa is reflected. According to Hagger et al. [59], the intention to follow social distancing during the pandemic, social norms, and moral obligations were important determinants in an actual practice. This implies that it is important for individuals to follow the guidelines suffeciently during a crisis, such as a pandemic situation, when the government and the media deliver messages to the public. It is also important to promote that it is beneficial to society [60].

The fourth factor of this instrument is the emotional dimension, including ‘If I have an abnormality in my body, I am worried about getting infected, so I am more careful’ and ‘I become anxious and careful whenever I think about the pandemic’. Emotion is a component of attitude, and an attitude is formed through positive or negative emotions about an object, issue, or action [61]. Therefore, the emotional dimension in this study can be seen as reflecting the feelings for a socially negative situation called a pandemic. This supports the findings of Lakhan, Agrawal, and Sharma [62] that pandemic situations can cause emotional problems while giving people mental distress, such as depression, anxiety, and stress. It also shows that attitudes are formed according to these psychological reactions.

The fifth factor of this instrument is the managerial dimension, including ‘I think proper exercise can help in reducing the chances of getting infected’ and ‘I think it is easy to fight against infection if I boost my immunity by ingesting nutrients in a balanced manner’, thereby showing the willingness of preventive action against a pandemic. The intentional dimension in this study supports the opinion that in a single-dimensional model of attitude, the mutual agreement among cognitive, emotional, and behavioral elements represents an attitude [61]. However, as research on this is insufficient, it is necessary to examine the variables that induce intention or influence intention in future studies, and to prepare an intervention program that can improve intention.

## 5. Limitations and Significances

This study is meaningful in that it has developed an instrument that can evaluate pandemic attitudes by reflecting various attributes that can represent people’s attitudes in pandemic situations. Through this, it is expected to contribute to the vitalization of research that can promote health by predicting and changing human behavior in a pandemic situation. This is used as a basis for the need for a protocol for health care professionals to reduce the burden of psychological stress, which is constantly increasing due to intervention activities or demands on people during the pandemic [63], to reduce the burden of workload in the future. It is inferred that this is possible. As a global pandemic causes mental health problems, it hinders policy makers and medical institutions making decisions in countries around the world in preparing pandemic response strategies [13]. Interventions through the measurement results of this tool will also be also helpful for experts related to infectious diseases. Furthermore, this study has developed an instrument for measuring pandemic attitude with verified reliability and validity by performing an exploratory factor analysis for the items on pandemic attitude, a confirmatory factor analysis on other subjects based on the results of the exploratory factor analysis to secure cross validity, and verifying the construct validity step by step. By accurately measuring the pandemic attitude of adults using the developed tool, it can be used as a tool for developing various interventions or policies and management strategies as in the current COVID-19 pandemic situation. For this purpose, reliability and validity were verified through an online survey of 390 general adults. In the future, repeated studies are needed to revalidate the tools for various groups by expanding the sample.

Regarding limitations of the study, first, as test-retest reliability verification was not performed in this study, a follow-up study to verify the stability of the tool is considered necessary. Second, the measurement tool developed in this study was developed for healthy general adults and has limitations in applying it to subjects with diseases or disabilities. Despite these limitations, it is expected that the developed tool will be used as useful data for reflecting the reality of the pandemic situation and preparing interventions, preventive activities, and policies by measuring the attitudes toward pandemics in adults.

As the only tool that can measure an epidemic attitude, the tool developed in this study needs to be refined continuously, and its validity and reliability need to be re-verified in the future. In addition, through this study, we propose a study to identify the variables that affect the epidemiological attitude and develop a conceptual model.

## 6. Conclusions

This study was to contribute to nursing research and nursing practice by developing an instrument with secured validity and reliability for measuring the pandemic attitudes of ordinary adults in Korea and by using it for the assessment, intervention, and evaluation of pandemic attitudes of ordinary adults. The developed instrument (A-PAS) is an instrument with verified validity and reliability, as well as the specificity of the pandemic situation reflected. Therefore, this instrument can be effectively used to assess and evaluate the attitudes of people in situations where pandemic and other infectious diseases develop, and the outcomes are expected to serve as fundamental data for preparing plans and interventions for disease prevention and health promotion.

## Figures and Tables

**Table 1:** Steps of development and evaluation of A-PAS.

Steps	Development of A-PAS	Evaluation of A-PAS
Procedures	- Development of preliminary items - Content validity - Face validity - Preliminary investigation	- Item analysis - Construct validity: exploratory factor analysis, confirmatory factor analysis - Convergent validity - Discriminant validity - Criterion validity - Factor naming - Reliability: internal consistency - Completion of the final instrument

**Table 2:** General characteristics of the study participants.

Characteristics	Categories	Data Set A (*n* = 170) *n* (%) or Mean (SD)	Data Set B (*n* = 220) *n* (%) or Mean (SD)
Gender	Male	77 (45.3)	109 (49.5)
	Female	93 (54.7)	111 (50.5)
Age		37.87 (10.47)	37.81 (11.52)
Marital status	Married	85 (50.6)	104 (47.3)
	Single	81 (47.6)	110 (50.0)
	Etc.	3 (1.8)	6 (2.7)
Religion	Protestantism	32 (18.8)	31 (14.1)
	Catholic	11 (6.5)	20 (9.1)
	Buddhism	15 (8.8)	28 (12.7)
	None	106 (62.4)	130 (59.1)
	Other	6 (3.5)	11 (5.0)
Job	Employee	77 (45.3)	91 (41.4)
	Official	6 (3.5)	10 (4.5)
	Self-employed	12 (7.1)	16 (7.3)
	Profession	15 (8.8)	18 (8.2)
	Service	16 (9.4)	11 (5.0)
	Other	44 (25.9)	74 (33.6)
Perceived economic level	Very bad	23 (13.5)	25 (11.4)
	Bad	57 (33.5)	76 (34.5)
	Normal	75 (44.2)	98 (44.6)
	Good	15 (8.8)	21 (9.5)
	Very good	0 (0)	0 (0)
Perceived health condition	Very bad	2 (1.2)	1 (0.5)
	Bad	27 (15.9)	40 (18.2)
	Normal	96 (56.4)	123 (55.9)
	Good	41 (24.1)	52 (23.6)
	Very good	4 (2.4)	4 (1.8)

**Table 3:** Corrected item-total correlation and factor loadings in EFA using data set A (*n* = 170).

Factors	Items No.	Cronbach’s α after Item Deleted	CIT	Factor Loading				
				Factor A1	Factor A2	Factor A3	Factor A4	Factor A5
Factor A1	Item24	0.92	0.64	0.79				
	Item23	0.92	0.59	0.76				
	Item15	0.92	0.48	0.66				
	Item13	0.92	0.68	0.64				
	Item9	0.92	0.58	0.60				
	Item14	0.92	0.61	0.54				
	Item16	0.92	0.60	0.49				
	Item3	0.92	0.50	0.41				
Factor A2	Item1	0.92	0.50		0.79			
	Item4	0.92	0.64		0.74			
	Item10	0.92	0.68		0.67			
	Item2	0.92	0.63		0.55			
	Item11	0.92	0.68		0.49			
	Item12	0.92	0.60		0.49			
	Item5	0.92	0.67		0.47			
Factor A3	Item18	0.92	0.46			0.69		
	Item22	0.92	0.57			0.65		
	Item25	0.93	0.33			0.62		
	Item17	0.92	0.54			0.60		
Factor A4	Item20	0.93	0.34				0.80	
	Item19	0.92	0.58				0.72	
	Item21	0.92	0.61				0.67	
Factor A5	Item7	0.92	0.40					0.88
	Item6	0.92	0.45					0.82
	Item8	0.92	0.62					0.47
Eigen value				4.37	3.88	2.56	2.19	2.17
Proportion of variance: total: 60.6% of variance				17.46	15.50	10.22	8.75	8.68
Cronbach’s a (total = 0.92)				0.86	0.86	0.69	0.75	0.75
Range of corrected item-total correlation				0.27~0.66	0.33~0.62	0.23~0.48	0.48~0.51	0.35~0.66
Mean (SD) (Total: 105.25 (10.94))				35.61 (3.85)	29.81 (3.86)	16.11 (5.17)	12.06 (1.67)	11.66 (9.99)

CIT = corrected item-total correlation.

**Table 4:** Analysis of construction validity.

	χ^2^(*p*)	df	CIMIN/df	RMSEA	RMR	SRMR	GFI	IFI	TLI	CFI
Model 1	2016.86 (*p* < 0.001)	190	2.46	0.08	0.06	0.09	0.81	0.85	0.83	0.85
Model 2	395.90 (*p* < 0.001)	160	2.47	0.03	0.03	0.06	0.84	0.89	0.89	0.89
Model 3	134.05 (*p* < 0.001)	120	1.12	0.02	0.02	0.04	0.94	0.99	0.99	0.99

**Table 5:** Analysis of convergent validity of items (*n* = 220).

Factors	Items	Standardized Estimates	Non-Standardized Estimate	S.E.	C.R.	*p*	AVE	CR
F1	Item24	0.72	1.00	-	-	-	0.59	0.92
	Item23	0.65	1.10	0.11	9.93	< 0.001		
	Item15	0.70	1.15	0.12	10.00	< 0.001		
	Item13	0.71	1.03	0.10	10.17	< 0.001		
	Item9	0.65	0.90	0.10	9.21	< 0.001		
	Item14	0.73	1.07	0.10	10.68	< 0.001		
	Item16	0.74	1.04	0.11	9.60	< 0.001		
	Item3	0.61	0.99	0.12	8.56	< 0.001		
F2	Item4	0.74	1.00	-	-	-	0.67	0.91
	Item10	0.72	0.96	0.09	10.21	< 0.001		
	Item2	0.72	1.11	0.10	11.11			
	Item12	0.72	1.06	0.11	10.03	< 0.001		
	Item5	0.75	1.10	0.10	10.56	< 0.001		
F3	Item22	0.70	1.00	-	-	-	0.54	0.70
	Item17	0.61	0.68	0.09	7.95			
F4	Item20	0.70	1.00	-	-	-	0.68	0.86
	Item19	0.73	1.01	0.12	8.28	< 0.001		
	Item21	0.81	0.94	0.11	8.37	< 0.001		
F5	Item7	0.71	1.00	-	-	-	0.73	0.84
	Item6	0.91	1.49	0.26	5.75	< 0.001		

**Table 6:** Discriminant validity of average variance extracted and 95% confidence interval in confirmatory factor analysis.

Variables	Φ^2^					AVE	Raykov’s *p*	Cronbach’ s α (Total)	
	Factor 1	Factor 2	Factor 3	Factor 4	Factor 5				
Factor 1	1					0.59	0.88	0.87	(0.92)
Factor 2	0.01	1				0.67	0.85	0.84	
Factor 3	0.01	0.08	1			0.54	0.60	0.61	
Factor 4	0.02	0.03	0.05	1		0.68	0.79	0.79	
Factor 5	0.01	0.04	0.08	0.06	1	0.73	0.80	0.78	
**Factor↔Factor**			**Φ**			**SE**	**Φ – 2 × SE**	**Φ + 2 × SE**	
1↔2			0.08			0.04	0	0.16	
1↔3			0.12			0.04	0.04	0.20	
1↔4			0.13			0.04	0.05	0.21	
1↔5			0.08			0.04	0	0.16	
2↔3			0.28			0.04	0.20	0.36	
2↔4			0.18			0.03	0.12	0.24	
2↔5			0.21			0.03	0.15	0.27	
3↔4			0.23			0.05	0.10	0.33	
3↔5			0.29			0.04	0.21	0.37	
4↔5			0.25			0.03	0.19	0.31	

**Table 7:** Adult Pandemic Attitude Scale (A-PAS).

Factor	Adult Pandemic Attitude Scale (A-PAS)
Personal Belief Attitude (8)	I think I should pay more attention to personal hygiene when there is a pandemic.
	The idea that it is ok for me alone to break the rules can be problematic.
	I think my health precedes religion or political beliefs.
	I think it is necessary to wear personal protective equipment (e.g., masks, etc.) when a pandemic occurs.
	I think the pandemic can have a serious impact on society overall.
	I think even a healthy person can get infected during a pandemic.
	I think that when a pandemic occurs, the lifestyle recommended by the government or local government should be followed.
	When a pandemic occurs, I avoid crowded places.
Knowledge Attitude (5)	In the event of a pandemic, I think I can get infected through contact with others.
	I can also get infected during a pandemic, so I should always be on the lookout.
	When a pandemic occurs, I try to obtain information from the mass media.
	I think social distancing is necessary when an outbreak of infectious disease occurs.
	I advise my family about infection and urge them to be careful when a pandemic occurs.
Normative Attitude (2)	I get angry when I see people who ignore government guidelines.
	I follow the guidelines provided by the government precisely because fake news or misinformation adversely affects pandemic control.
Emotional Attitude (3)	If I have an abnormality in my body, I am worried about getting infected, so I am more careful.
	I become anxious and cautious whenever I think about the pandemic.
	I take precautions ahead of time if I experience symptoms of infection similar to those of the pandemic.
Managerial Attitude (2)	I think proper exercise can help in reducing the chances of getting infected.
	I think it is easy to fight against infection if I boost my immunity by ingesting nutrients in a balanced manner.
Scoring of A-PAS The A-PAS is a self-report instrument with a 5-point Likert scale to assess the level of pandemic attitude. All items are rated on a 5-point scale, namely, “Strongly disagree (1)”, “Disagree (2)”, “Usually (3)”, “Agree (4)”, and “Strongly agree (5)”.	

## Data Availability

No new data were created or analyzed in this study. Data sharing is not applicable to this article.
